# User fee exemptions and equity in access to caesarean sections: an analysis of patient survey data in Mali

**DOI:** 10.1186/1475-9276-11-49

**Published:** 2012-08-29

**Authors:** Marianne El-Khoury, Laurel Hatt, Timothee Gandaho

**Affiliations:** 1 Associate/Economist, Abt Associates Inc, 4550 Montgomery Ave, Suite 800 North, Bethesda, MD 20814, USA; 2 Senior Associate/Health Economist, Abt Associates Inc, 4550 Montgomery Ave, Suite 800 North, Bethesda, MD 20814, USA; 3 Senior Advisor & Team Leader, Abt Associates, Bamako, MALI

**Keywords:** User fees, Equity, Caesarean sections, Mali, Access

## Abstract

**Introduction:**

Little rigorous evidence exists on how health service utilization varies across socioeconomic groups after a user fee exemption policy has been implemented, and the evidence that does exist is mixed. In this paper, we estimate the distribution of caesarean section deliveries across socioeconomic groups following Mali’s implementation of a fee exemption policy for caesareans in 2005.

**Methods:**

We conducted a patient survey in 2010 to collect socioeconomic data from 2,477 women who had caesareans in a representative sample of 25 facilities across all regions of Mali. We used these data along with data from the most recent Demographic and Health Survey to construct a wealth index and classify women into population-based wealth groupings. We compared the wealth distribution of women delivering via caesarean section to that of a nationally representative sample of women giving birth.

**Results:**

We found that wealthier women make up a disproportionate share of those having free caesareans, five years after implementation of the fee exemption policy. Women in the richest two quintiles accounted for 58 percent of all caesareans, while women in the poorest two quintiles accounted for 27 percent of all caesareans. Fewer women in the poorest two-fifths of the population are receiving caesareans than what we would expect given their share in the population of women giving birth.

**Conclusions:**

While fee exemptions remove important financial barriers to accessing priority maternal health services, they are insufficient to ensure equal access among wealth groups.

## Introduction

In the late 1980s, many countries in Africa introduced user fees in public sector health facilities as a way for under-resourced facilities to secure the financing needed to provide basic health care. User fees are nominal out-of-pocket charges for health services and are meant to provide revenue to procure drugs and pay for items such as fuel for emergency transport or cleaning supplies. In recent years, user fee policies have come under high scrutiny. Fees have been increasingly regarded as a prohibitive barrier to access, especially access to essential services among poor populations who often choose to delay or forgo seeking care to prevent further impoverishment [1,2].

Consequently, some countries have abolished user fees for a number of preventive health services (for example, South Africa in 1994, Ghana in 1996 and 2008, Uganda in 2001, Mali in 2005, Zambia and Burundi in 2006, Burkina Faso in 2006, and Benin in 2009). Many studies have shown that overall utilization of services increased following the reduction or removal of user fees [1,3-5]. However, little rigorous evidence exists on how utilization of services varies across socioeconomic groups after a fee exemption policy has been implemented, and the evidence that does exist is mixed. In Uganda, fee exemptions appear to have increased utilization of outpatient services among the poor [3], whereas exemptions in southeast Nigeria were shown to have mostly benefited richer households [6]. A detailed review of available research on user fees [1] concluded that utilization of fee-exempt health services among the poor is limited by additional constraints such as informational, geographical, and cultural barriers to care.

In this paper, we estimate the distribution of caesarean section deliveries across socioeconomic groups in Mali five years following the introduction of a fee exemption policy. Using a methodology adapted from Pitchforth et al. [7], we measure the distribution of women delivering via caesarean sections across socioeconomic groups and compare this to the distribution of all women giving birth. Our results provide evidence that fee exemptions alone are not sufficient to achieve equity in use of caesarean section deliveries across socioeconomic groups.

### The caesarean section fee exemption policy in Mali

The maternal mortality ratio in Mali is among the highest in the world (464 per 100,000 live births) [8] and access to maternal health services is low, especially in rural areas. Skilled birth attendance and lifesaving obstetric procedures such as caesarean sections are considered critical interventions for safe motherhood, as they allow a timely response to potentially fatal emergencies [9,10]. The affordability of obstetric care thus has large implications for maternal and neonatal survival and well-being through its effect on access to care at delivery.

In Mali, population-based caesarean rates in 2005 were estimated at less than 1.6 percent of births [8], well below the 5 to 15 percent range considered essential for safe motherhood by the World Health Organization (WHO) [11]. To address this large unmet need for emergency obstetric care, the government of Mali in 2005 removed user fees for caesarean sections in public sector facilities throughout all regions of the country. Community health clinics or health posts are the individual’s first level of contact with the health system in Mali. These clinics only perform normal deliveries. Caesarean sections are performed in health centers and regional hospitals located at the district level, and in large hospitals in the capital city, Bamako. The exemption policy removed charges to patients for the direct costs of the caesarean procedure, including for pre-operative examinations, surgery, post-operative treatment, and laboratory tests. User fees are still charged for normal deliveries and antenatal care at health facilities.

The initiative received widespread support inside Mali, with high expectations for reducing maternal mortality. The average price to a patient of a caesarean section, which ranged between FCFA 47,400 and FCFA 68,000 pre-policy (approximately US$ 95–136), was significantly reduced to FCFA 400 – 4,800 (or approximately US$ 0.8-10) [12-15]. Since then, caesarean rates in Mali have been steadily rising in all regions of the country – from an estimated 1.9% in 2006 to 2.3% in 2009 according to the Health Management Information System in Mali [16]. This increase in utilization is in line with experiences in other countries that have implemented fee exemptions for preventive or curative health care services [2-5,17]. The proportion of caesarean procedures that resulted in maternal and neonatal deaths also declined, possibly because delays in initiating surgery were reduced with the introduction of the policy [16].

## Methods

### Overview

We conducted a facility-based patient survey in 2010 in a nationally representative sample of 16 public sector health centers and 9 hospitals that offer caesarean services. The survey collected data on a small set of demographic and socioeconomic variables from 2,477 women who had caesareans in these facilities over an 8-month period. We then constructed a wealth index for each patient in the survey using a set of household asset variables. Next, we used data from the most recent (2006) Demographic and Health Survey (DHS) in Mali to classify these caesarean patients into population-based wealth groupings. Finally, we compared the wealth distribution of caesarean patients to that of all women giving birth in the population.

### The 2010 patient survey

To select the sample of health facilities for the patient survey, we first stratified all public sector health centers that perform caesareans by region. In each of the eight regions in Mali and the District of Bamako, we further stratified facilities into two groups: those with caesarean rates above the median for the region (as of 2008) and those below the median for the region. We then randomly selected one health center from each group (n = 16). At the time of the sampling design, none of the health centers in the northeastern region of Kidal had registered any caesarean procedure, so we did not select a center from this region. We also included with certainty the regional hospital in each region and one tertiary hospital in Bamako (n = 9). The final sample size was 25 facilities. During an 8-month period (February – September 2010), we surveyed every woman who had a caesarean procedure in the 25 sampled facilities and completed 2,477 interviews. Three women refused to participate in the survey.

The sample was limited to public sector facilities, as the caesarean policy was only implemented in public facilities and only 2.4 percent of all deliveries in Mali occur in private facilities [8]. Most deliveries either take place at home or in a public facility.

Since every woman who had a caesarean in a sampled facility was included in the survey, respondents in the same facility were assigned the same sampling weight. Sampling weights were calculated as the inverse of the probability of a facility being selected into the sample and were adjusted by the size of each facility (in terms of the number of deliveries per year). Since only three women refused to participate in the survey, no non-response adjustment was made.

The research protocol and survey instrument were approved by the Abt Associates Institutional Review Board and the national ethics committee in Mali (known as the *Comité National d’Ethique pour la Santé et les Sciences de la Vie)*. Every person associated with the study (data collection, translation, information handling) signed a confidentiality agreement to maintain confidentiality and anonymity of the data. All interviewees were asked to provide verbal consent before participating and were free to decline to participate. The questionnaire asked for basic information about the patient such as her age and number of children, as well as selected indicators of household asset ownership. These included the main floor material of her dwelling, source of drinking water, type of cooking fuel used, whether the household owns a bicycle, and whether the household owns a television.

### The 2006 DHS

The most recent publicly-available standard DHS for Mali was conducted in 2006, with data reported around the same time as the caesarean policy was introduced. The individual female dataset includes a nationally representative sample of 14,383 women of reproductive age [18]. It includes a range of indicators on maternal health as well as indicators on household ownership of assets that are aggregated to form a wealth index. Wealth is proxied by household ownership of selected assets that vary across income groups [19]. Following Filmer and Pritchett [20], the DHS uses Principal Component Analysis to assign weights and aggregate these asset variables into a wealth index. Households are then assigned to equally-sized quintiles ranging from the poorest to the wealthiest. The wealth index from the DHS served as the “gold standard” population wealth index for our analysis.

### Creating a ‘proxy’ wealth index in the DHS

We adapted a methodology first published by Pitchforth et al. in 2007 [7] that aimed to facilitate rapid collection of socioeconomic data from exiting patients. The authors developed an approach to calculate a wealth index using a small number of indicators collected at the facility level [7]. Using the individual female dataset of the Mali DHS, we identified the subset of five variables used in constructing the full DHS wealth index that showed the greatest variation across wealth quintiles in that survey. We regressed the full DHS wealth index (constructed using 12 variables) on dummies for the five selected variables. The fitted values from this regression constituted our “proxy” wealth index. The regression had an R^2^ value of 0.8, indicating that the five selected variables explained 80 percent of the variability in the full DHS wealth index. Next, we used the proxy wealth index to rank women in the DHS sample, grouped them into quintiles, and identified cutoff values of the proxy index for each quintile.

### Creating a wealth index in the 2010 patient survey

Questions about these five selected assets were included in our patient survey of post-caesarean women. Unlike Pitchforth et al. who used researcher-assigned weights for the assets in their patient survey, we used the estimated coefficients from the DHS regression described above as weights to construct a comparable wealth index. We then used the cutoff values from the DHS proxy index to classify our caesarean survey respondents into nationally representative wealth quintiles.

### Comparing distributions

We compare the wealth distribution of the caesarean sample (2010 patient survey) to that of the most recent sample of deliveries (2006 DHS). We computed the ratio of the share of caesarean to that of deliveries in each wealth group to highlight any disparities between the two distributions. We calculated standard errors for the ratios and report confidence intervals at the 95% level.

### Sensitivity checks

Including only five asset variables in a wealth index may limit the ability of the index to discriminate finely among individuals with similar wealth status. We therefore performed checks to test the sensitivity of the results to different values of the cutoff points that classify women into wealth groups. We used the cumulative distribution of the proxy index in the larger population-based DHS dataset to identify “natural” cutoff points that occur around peak values of the index, and compared the distribution of deliveries and caesareans based on these new wealth groupings.

Given the availability of health facilities, qualified staff, and transportation, access to health services in Bamako is typically higher than in other regions. Furthermore, the DHS wealth index does not reflect much socioeconomic variation for Bamako as it categorizes approximately 97 percent of residents of Bamako as being among the richest 40 percent of the population. Data on additional asset variables that would make it possible to capture wealth variations within Bamako were not collected in the 2006 DHS, and thus not collected in our patient survey. To exclude the possibility of Bamako driving the main results, we reanalyzed the data excluding Bamako from the sample.

## Results

Table 1 compares the socioeconomic and demographic characteristics of our sample of women who underwent caesareans with those of women in the DHS sample who had given birth in the year preceding the survey. On average, women in the caesarean sample had slightly fewer children, higher educational attainment, and better dwelling characteristics, and were more likely to own a television or bicycle.

**Table 1:** Socioeconomic and demographic characteristics in two study samples

Characteristics*		**2010 patient survey (sample of caesareans)**	**2006 DHS survey (sample of deliveries)**
**N**		2,477	3,155
**Age**	Range	13-51	15-49
	Average	25.8	26.9
**Parity**	Range	1-15	1-14
	Average	3.6	4.2
**Education level**	None	73%	84%
	Primary	18%	11%
	Secondary	7%	5%
	Higher education	2%	0.3%
**Main floor material**	Dirt/sand	54%	61%
	Dung	7%	17%
	Carpet	4%	2%
	Cement	35%	20%
	Other (tile, parquet)	0.2%	1%
**Main fuel source**	Wood	71%	85%
	Charcoal	28%	13%
	Electricity, LPG	1%	0.1%
	Other	1%	2%
**Drinking water source**	Unprotected well	30%	43%
	Protected well	28%	30%
	Piped to yard/plot	9%	3%
	Piped into dwelling	4%	5%
	Public tap/standpipe	27%	14%
	Other	1%	4%
**Own television**		50%	24%
**Own bicycle**		57%	53%

*weighted.

**Figure 1:**
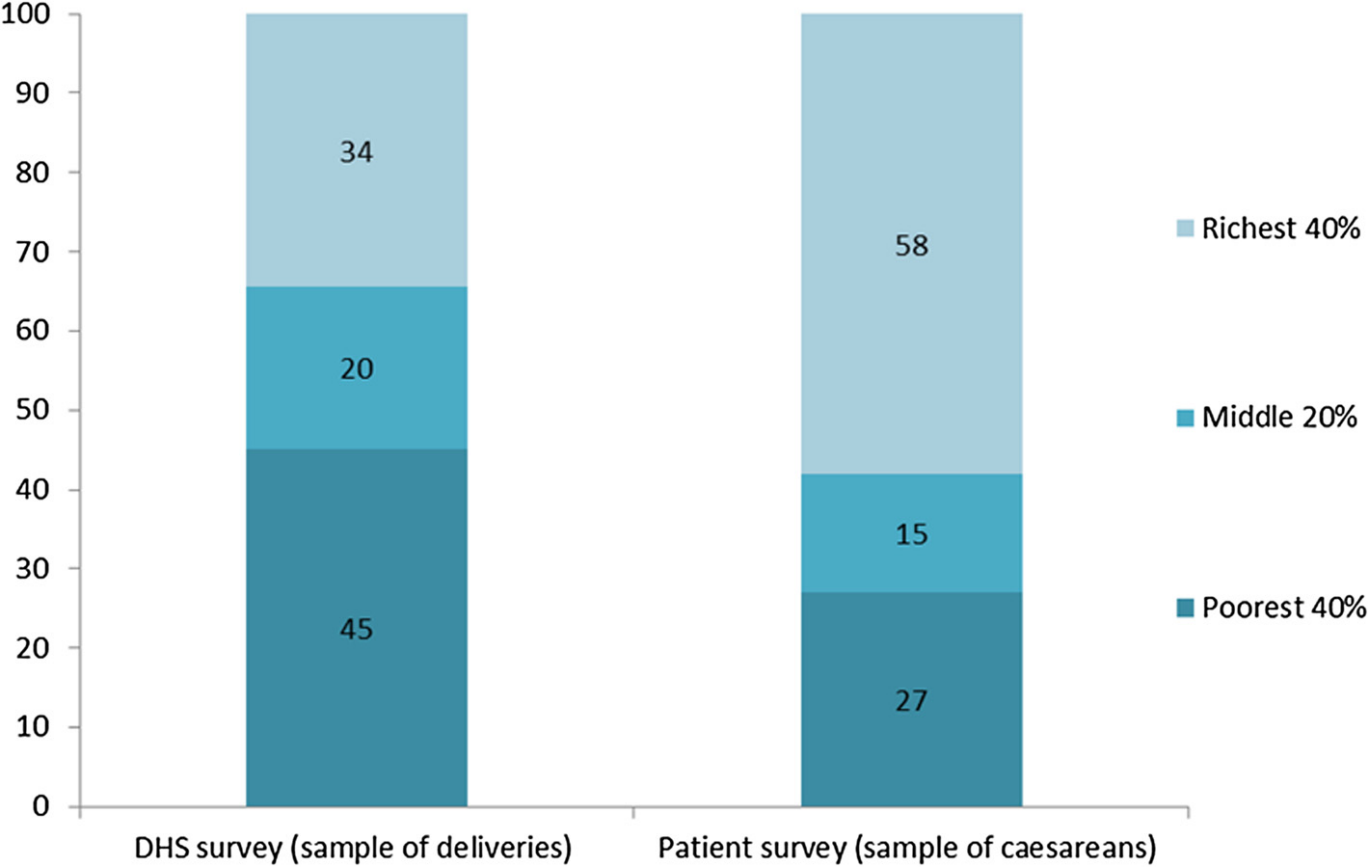
Wealth distribution of deliveries and caesareans, in percent.

Figure 1 shows the distribution of the DHS sample of deliveries and the caesarean survey sample according to nationally representative wealth groups. Women in the caesarean sample are on average wealthier than the sample of all women who gave birth: approximately 58 percent of caesareans occurred among women in the richest 40 percent of the population, compared with 34 percent of deliveries. Put differently, the share of caesareans in the richest 40 percent is more than one and a half times (a factor of 1.67) the share of deliveries in that wealth group (Table 2). In contrast, only 27 percent of caesareans occurred among women in the poorest 40 percent of the population, compared with 45 percent of deliveries. If access to caesareans were equal among wealth groups, we would expect the wealth distribution of women receiving caesareans to be the same as the wealth distribution of women who had given birth. Our analysis shows that fewer women in the poorest two-fifths of the population are receiving caesareans than what we would expect given their share in the population of women giving birth.

**Table 2:** Wealth distribution of deliveries and caesareans

Wealth groups*	**Column A: 2006 DHS survey (sample of deliveries)**	**Column B: 2010 patient survey (sample of caesareans)**	Ratio of column B to column A [95% CI]**
**Richest 40%**	34.4%	57.6%	1.67 [1.43-1.91]
**Middle 20%**	20.4%	15.4%	0.75 [0.61-0.89]
**Poorest 40%**	45.1%	27.0%	0.59 [0.47-0.71]

* For simplicity, we combined the ‘poorest’ and the ‘poorer’ quintiles (poorest 40 percent), and the ‘richest’ and the ‘richer’ quintiles (richest 40 percent).

** 95-percent confidence intervals (CI) in brackets.

### Sensitivity checks

We performed checks to test the sensitivity of the results to different values of the cutoff points that classify women into wealth groups. We used the cumulative distribution of the proxy index in the larger population-based DHS dataset and identified four “natural” groupings with cutoff points at the cumulative sums of 14, 27, and 60 percent of the index’s distribution. We compared the distribution of deliveries and caesareans based on these new wealth groupings and found similar results: the distribution of caesareans was consistently skewed to the wealthier group.

We found similar results in the subsample of women from regions excluding Bamako (Table 3): women in the richest two quintiles accounted for approximately 48 percent of all caesareans, compared to 27 percent of deliveries. Women in the poorest two quintiles accounted for 33 percent of all caesareans, compared to 50 percent of deliveries.

**Table 3:** Wealth distribution of deliveries and caesareans, excluding bamako

**Wealth groups**	**Column A: 2006 DHS survey (sample of deliveries)**	**Column B: 2010 patient survey (sample of caesareans)**	Ratio of column B to column A [95% CI]**
**Richest 40%**	27.5%	48.3%	1.76 [1.45-2.07]
**Middle 20%**	22.4%	18.3%	0.82 [0.66-0.98]
**Poorest 40%**	50.1%	33.3%	0.66 [0.56-0.76]

* 95-percent confidence intervals (CI) in brackets.

## Discussion

In this study we used an innovative method to estimate the socioeconomic distribution of women having caesareans five years after implementation of a user fee exemption policy. The results of the analysis show that wealthier women in Mali make up a disproportionate share of those having free caesareans. It is important to note that it is not the purpose of this paper to evaluate the extent to which the caesarean fee exemption policy has improved equity in access to caesarean sections; data necessary to answer this question do not exist. Rather, the paper is concerned with examining whether substantial inequalities in access to an essential health service persist, despite the presence of a policy primarily instituted to remove a significant access barrier among the poor.

Our findings are consistent with the results of a larger qualitative study [16] that details the challenges of policy implementation and the community’s perceptions of remaining barriers to caesarean access. That study found that transportation costs and difficult road conditions are seen as significant barriers to reaching facilities and accessing caesarean services. Transport costs are typically most prohibitive among the poorer wealth groups who are more likely to live in remote rural areas and have limited access to a health facility. At the same time, user fees are still charged for normal deliveries, thus wealthier women who can afford to go to a health facility in expectation of a normal delivery are more likely to have access to a caesarean should complications arise during labor. Poor women delivering at home face transportation barriers that limit their access to facility-based services. In addition, it appears that supply-side constraints may have further reduced access among the poorest women. Health care providers reported that the government-provided caesarean kits were frequently incomplete or contained expired medicines [16]. Wealthier households can afford to pay for any extra medication needed and are thus more likely to have access to the service. The qualitative study also found that cultural factors are associated with low utilization of facility-based maternal health services. Illiteracy and low awareness of the benefits of medically-assisted deliveries, typically more common among low-income women, continue to correlate with home-based deliveries. Finally, antenatal care services were not exempted from user fees in Mali. As a result, wealthier women who receive more antenatal care [8] may have been more likely to learn about the caesarean policy during their visits than other women were.

This study provides evidence that inequalities in access to services across wealth groups persist despite the presence of fee exemption policies that were primarily implemented to remove a prohibitive financial barrier. It is plausible and even likely, however, that access among the poor improved from an even more inequitable baseline distribution as a result of the policy. The lack of a comparable dataset prior to policy implementation does not allow further exploration of this hypothesis.

Our study has a number of limitations. First, the caesarean data and the data on deliveries were collected four years apart, which could introduce some bias if the population’s wealth improved greatly during that time period. The 2006 DHS dataset, however, is the most recent dataset available that shows the distribution of deliveries across socioeconomic groups. In the absence of other data and until the next standard DHS results are released, the approach proposed in this study produces useful information for researchers and policymakers. Second, including only five asset variables in a wealth index limits the ability of the index to discriminate finely among individuals with similar wealth status. We primarily selected a limited number of variables in order to facilitate quick survey administration to patients in health facilities. However, the high R^2^ value in the regression of the DHS wealth index on these five asset variables and the sensitivity checks we conducted give us confidence that the index captures the bulk of the variability of the more comprehensive DHS index.

## Conclusions

When several African countries decided to remove user fees for high-priority health services in recent years, the policies were perceived as a step that could reduce prohibitive financial barriers and encourage service utilization among previously excluded populations. Indeed, it is encouraging that caesarean sections rates in Mali appear to have increased since the introduction of the policy. However, this study indicates that wealthier women make up a disproportionate share of those having free caesareans. While fee exemptions remove important treatment-related financial barriers, other barriers – both financial and non-financial – still persist for poor women in Mali. Unless simultaneously and directly addressed, these barriers will impede realizing the full potential of the policy. In addition, careful planning is needed to ensure that the government can continue to prioritize these subsidies, especially as population uptake continues to increase.

## Abbreviations

DHS: Demographic and Health Survey; USAID: United States Agency for International Development; WHO: World Health Organization.

## Competing interests

The authors declare that they have no competing interests. The opinions expressed herein are the authors’ and do not necessarily reflect the views of Abt Associates Inc. or the U.S. Agency for International Development (USAID). All errors remain the responsibility of the authors.

## Authors’ contributions

MEK analyzed and interpreted the data and drafted the manuscript. LH made significant contributions to the data interpretation and analysis and revised the manuscript critically for important intellectual content. TG participated in the original design of the study and the coordination of data collection in the field and was involved in revising the manuscript. All authors read and approved the final manuscript.
